# HIV in Nepal: Is the Violent Conflict Fuelling the Epidemic?

**DOI:** 10.1371/journal.pmed.0020216

**Published:** 2005-07-19

**Authors:** Sonal Singh, Edward Mills, Steven Honeyman, Bal Krishna Suvedi, Nur Prasad Pant

## Abstract

Singh and colleagues discuss the HIV epidemic in Nepal, including the local and international response from the health and development community.

HIV/AIDS has reached alarming proportions in Southeast Asia [1]. The magnitude of the epidemic is projected to exceed that of sub-Saharan Africa in the 21st century [2]. More than 7 million South Asians are currently infected with HIV [3], nearly 5 million of whom are in India [3].

Nepal has had a comparatively lower prevalence of HIV/AIDS compared to other countries in Southeast Asia. Seasonal migration and sexual trafficking across a porous Indian border [4], fuelled by a bloody Maoist conflict (see Sidebar), has raised Nepal's HIV prevalence to the second highest in the region after India.

In this essay, we characterize the current HIV epidemic in Nepal, look at the ways in which the conflict may be fuelling the infection rate, and discuss the current local and international response from the health and development community. We argue that there is currently a short window of opportunity to take action to control the HIV epidemic in Nepal—and that inaction will lead to HIV becoming the biggest killer of the young and middle-aged in the next decade.

## The Current HIV Epidemic in Nepal

The first case of AIDS was reported in Nepal in 1998 [5]. Most cases of HIV infection in Nepal are HIV-1, although HIV-2 was also recently reported [6]. As of February 2005, the National Center for AIDS and STD Control in Nepal reported that there were 4,755 HIV-positive people and 856 confirmed cases of AIDS in Nepal [7]. However, because of the poor surveillance systems and the lack of access to quality voluntary counselling and testing services coupled with antiretroviral treatment, these prevalence figures are likely to be a gross under-estimate [2].

**Violent Conflict in Nepal**

Nepal is one of the poorest countries in the world. Rebels, led by the Communist Party of Nepal (Maoist), have been fighting a bloody war with the government forces, led by the Royal Nepalese Army, since 1996. Originating from the western heartlands of Nepal, which were the least economically developed and most inaccessible regions of the country, the conflict has spread to nearly all of the 75 districts of Nepal. It has claimed more than 10,000 lives, led to widespread destruction of infrastructure, and disrupted the flow of essential supplies. Both sides have been accused of serious human rights violations. Violent clashes have escalated since the king seized power in February 2005, ousting his appointed prime minister and imposing a state of emergency [43].

A dramatically higher estimate comes from UNAIDS, which estimated that 62,000 out of a population of 24.1 million in Nepal were living with HIV/AIDS in 2003 [8]. One in 200 (0.5%) people aged 15–49 years are living with HIV/AIDS in Nepal [8]. About 30% of those infected are female [8]. WHO/UNAIDS estimates that 940 children are living with HIV and that nearly 13,000 children were orphaned in Nepal due to AIDS at the end of 2003 [5]. The prevalence in the general population may still be low, but it masks an increasing prevalence in several risk groups—the prevalence of HIV/AIDS consistently exceeds 5% in injecting drug users, commercial sex workers, and migrant workers [5].

## High-Risk Groups

Among injecting drug users—estimated to be about 30,000 in Nepal—about 40% are HIV-positive [9]. Needle sharing and risky sexual behaviour is common in this group. The figures are particularly alarming in Kathmandu, the capital city, where nearly 68% of injecting drug users are HIV-positive [10]. A subtype C virus with restricted genetic diversity is thought to have caused this epidemic in Kathmandu [11]. Concomitant hepatitis C infection is a contributing factor to the rapidity and severity of disease progression in injecting drug users, and 94% of users in Kathmandu have tested positive for hepatitis C [12].

The predominant mode of transmission of HIV in Nepal is heterosexual contact with commercial sex workers. HIV prevalence rates are about 4% among sex workers in the Terai regions of Nepal and about 1.5% in their clients (which is more than five times the national average prevalence) [13]. In Kathmandu, nearly 17% of sex workers are HIV-positive [8]. There are about 25,000 sex workers in Kathmandu [4] and an estimated 200,000 Nepalese women working in Indian brothels. About 5,000 to 10,000 Nepalese sex workers are trafficked every year [4], numbers that are likely to increase as a result of the conflict. One striking estimate is that nearly 70% of sex workers returning from India are HIV-positive [8].

As in India, a major contributor to the spread of HIV in Nepal has been Nepal's mobile population, including truckers and migrant workers. About half of the estimated cases of HIV are in 29 highway districts of Nepal [14]. A 1999 survey indicated that 75% of the truckers surveyed had had sex with a sex worker and that only 70% of the truckers had used a condom during their last sexual encounter [15].

Men who have sex with men still account for only a small proportion of those affected with HIV in Nepal. But the recent detention and subsequent release of 39 male transvestite members of the Blue Diamond Society, a local organization that provides sexual health, HIV/AIDS, and advocacy services to sexual minorities, highlights the challenges faced by sexual minorities in Nepal [16].

## The Role of Conflict in Fuelling the Epidemic

Several factors contribute to the propagation of HIV in times of conflict (Box 1) [17]. While accurate numbers are hard to come by, the recent conflict may have contributed to the propagation of HIV/AIDS by fuelling displacement [18]. As the insurgency drags on, seasonal and long-term migration of labourers to neighbouring countries, such as India, is becoming critical to the economic survival of many households. Young men have left the country “en masse” for fear of execution and migrated to the high-prevalence areas in India. UNAIDS estimates at least 10% of the 2 million to 3 million Nepalese migrant workers in India are HIV-positive [19]. These men are now infecting spouses and others in many parts of the country. By pushing rural residents from war-torn areas to the capital, Kathmandu, the conflict may have helped spread HIV/AIDS. [Fig pmed-0020216-g001]

**Box 1. Factors Contributing to the Propagation of HIV in Times of Conflict**

- Increased levels of commercial sex
- Breakdown of vital services in health and education
- Decreased availability and use of reproductive health services
- Decreased use of means to prevent HIV transmission
- Increased population mixing following displacement, which may promote high-risk behaviour
- Fragmentation of families
- Sexual violence
- Stigmatization and discrimination related to HIV

**Figure pmed-0020216-g001:**
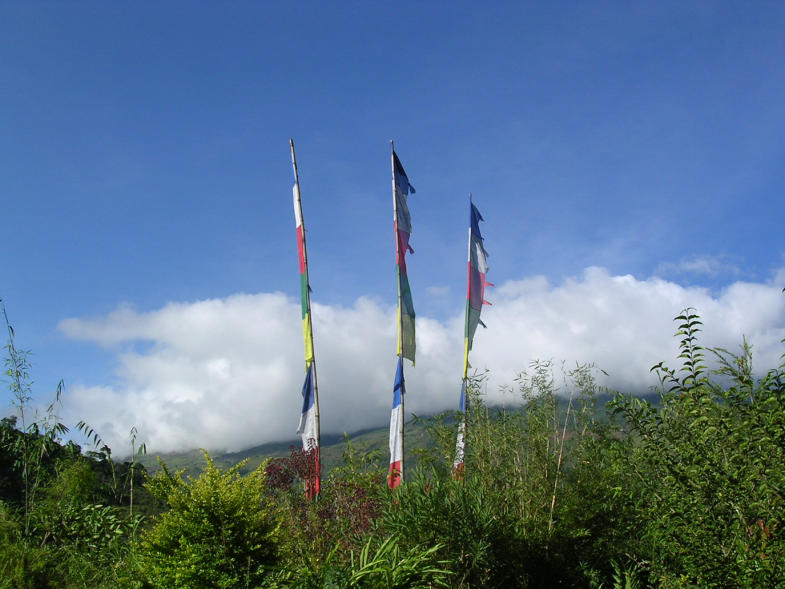
Prayer flags in the valley of Katmandu, Nepal

Some 200,000 to 400,000 people may have been displaced in Nepal since the beginning of the conflict [20]. Migrations within the country and internationally, coupled with a general lack of awareness and knowledge about risk factors, is likely to have contributed to the propagation of the epidemic. The far western regions of the country, which are less economically developed and which are the hotbed of the current insurgency, are more prone to migration. These regions have one of the fastest growth rates of HIV in South Asia.

Migrants are more likely to practice high-risk sexual behaviour [21]. About 8% of migrant male workers returning from Mumbai and examined in western Nepal were infected with HIV [22]. The epidemiological impact on women in migrant communities has yet to be realized, as only 0.5% of 900 women in the Kailali district in western Nepal were HIV-positive, although more than 30% had some form of STD [23].

Sex traffickers have shifted their trade from Sindhupalchowk and Nuwakot in central Nepal to Rukum and Rolpa (the hotbeds of the insurgency) in the mid-west, taking undue advantage of the socio-economic conditions borne by conflict and violence. The low socio-economic status of women along with the current conflict makes the women in this region particularly vulnerable [24]. Save the Children Norway's recent study, *Impacts of Armed Conflict Pushing Women and Girls Into Sexual Abuse and Sex Trade*, revealed that about 19% of female sex workers stated they had entered the sex trade directly because of the conflict [25].

While the number of people infected has risen, HIV prevention and awareness work has declined in Nepal as a result of the conflict. Save the Children's work in the Accham district of western Nepal has been hindered by fighting between the Maoist rebels and government forces since early 2002 [26]. Offices of nongovernmental organizations (NGOs) have been burned, and volunteers are afraid to work [26]. Médecins Sans Frontières was forced to curtail its activities last year in Jumla, one of the poorest districts in the Midwest, due to the conflict. Local activists fear that the situation will continue to deteriorate if NGOs are not allowed to work in Jumla. People living with HIV/AIDS in rural Nepal are desperate for care and support.

## Condom Use

In a study of young men in Nepal in 2001, only 42% of men used a condom consistently [27]. The vast majority of young men, about 80%, who had sex with non-regular partners felt they were not at risk of contracting any form of sexually transmitted disease or HIV [27]. The reasons for non-use included fear of losing sexual pleasure, embarrassment over buying condoms, and a belief that careful selection of partners offers sufficient protection [27]. Monitoring of behavioural trends has shown that condom use during the last sexual encounter as reported by female sex workers has markedly increased from 63% in 1998 to 90% in 2002 [28].

However, consistent use of condoms as reported by female sex workers (54%) still needs improvement [28]. Many female sex workers are young children and lack the power to negotiate safe sex [29]. Many of the young girls are considered free of disease by clients who see no need to use condoms. These girls command a high price as “virgins” for brothel owners who do not want to disturb the situation by requiring condom use [29]. Further, the weakening of traditional socio-cultural constraints in times of conflict makes the girls more prone to sexual abuse and exploitation and more likely to engage in high-risk sexual behaviour.

## The Local and International Response to the Epidemic

Nepal's National Center for AIDS and STD Control has received support from WHO, UNAIDS, UNDP, and USAID. But it lacks substantive funding to complete the necessary studies and interventions that are the key to HIV control. National efforts aimed at awareness of HIV are complicated by the ethnic diversity of Nepal, where some 75 different ethnic groups exist, speaking more than 50 languages.

A network of about 1,600 NGOs is now working on HIV/AIDS in Nepal. Local organisations are doing their best to reach out to those living with HIV/AIDS, but due to the urban-centric nature of most funding, funds available for the rural areas are scarce. Most donor representatives lack direct knowledge of the rural areas and rely on the instructions provided by NGOs in Kathmandu to dispense funds.

Foreign aid, which accounts for nearly 60% of Nepal's development budget, may have paradoxically contributed to lopsided development in Nepal. While aid money has favoured urban development and centralized power, the rural–urban gap has widened over the years. In Nepal, weak linkages between urban and rural areas, and lack of roads, communications, infrastructure, and appropriate skills among the rural poor mean that this urban bias has led to centralization of effective power on the one hand and maintenance of the economic, social, and political status quo on the other [30]. Urban biases inevitably play a deterrent role, discouraging poor patients from seeking help. The poor see very little of the aid money, since most of it is used for prevention, information, and awareness in urban centres rather than for care and support in rural areas.

In our experience, some donors consider HIV care and support to be too expensive to fund, arguing that Nepal lacks the kind of infrastructure—clinics, district hospitals, and distribution units—needed to provide effective antiretroviral treatment, and that antiretrovirals are a priority only in countries with a high prevalence of HIV/AIDS (1% or more) in the general population [31].

Harm-reduction interventions have been shown to slow the course of HIV among intravenous drug users (IDUs) in many developed countries [32], but in Nepal the concept of harm reduction is still new. Few harm-reduction programs are government-supported or integrated into mainstream service delivery. Organisations such as the Lifesaving and Lifegiving Society, a street-based NGO established in Nepal in 1991, have been providing education, counselling, and primary health care—as well as bleach, sterile water, condoms, and new needles and syringes—to IDUs to lower their risk of acquiring blood-borne diseases [33]. The prevalence of HIV infection among IDUs who were in regular contact with the program from 1991 to 1994 was low, at 1.6% [33].

However successful, these programs have not reached the border zones with India where HIV infection has risen dramatically among IDUs. This dramatic rise is not surprising since research has shown that cross-border drug-use patterns in areas of Nepal bordering India are particularly conducive to risky needle sharing [34]. Unlike IDUs in other Nepalese towns, very few of the IDUs in border towns belong to stable “injecting groups.” Sharing of contaminated injecting equipment in border towns is widespread, in part because of the makeshift arrangements in which the cross-border injecting takes place. Users often share their small amounts of money to buy drugs. Sexual intercourse with casual partners occurs, with inconsistent condom use [34]. Effective intervention would therefore require complex cross-border collaborative efforts [34].

People living with HIV/AIDS are stigmatized and face discrimination at all societal levels—in the community, at health facilities, and, most importantly, within the family [35]. In a recent survey by CARE-Nepal, almost half of those who came to the voluntary counselling and testing centre at the Doti District Hospital in Silgadhi, a conflict-affected area, during June–July 2004 tested positive for HIV, and almost all of those tested positive were widows in their twenties and thirties [35]. About 60% of them were breast-feeding their infants. These young widows faced rejection from their families, discrimination at work, and difficulty in coping with their life circumstances [35].

One important development in recent years has been within the area of condom promotion for HIV/AIDS prevention. A national research study found that 76.6% of retail outlets surveyed had never sold a condom in 2002 [36]. Population Services International began a national condom promotion program in early 2002 [37] using a three-pronged approach: (1) a national media campaign promoting condoms (Figure 1); (2) increased widespread condom availability within the private sector to destigmatize condom use; and (3) targeted condom promotion to high-risk groups such as sex workers [38]. The impact of such efforts on condom sales has been dramatic. Total sale of condoms jumped from 11.9 million units in 2002 to 23.1 million units in 2004, and sales continue to climb [39]. Creating long-term behaviour change and making condoms accessible in the private sector and affordable through subsidy to high-risk groups such as female sex workers appears to be increasing the uptake of condoms in Nepal.

**Figure 1:**
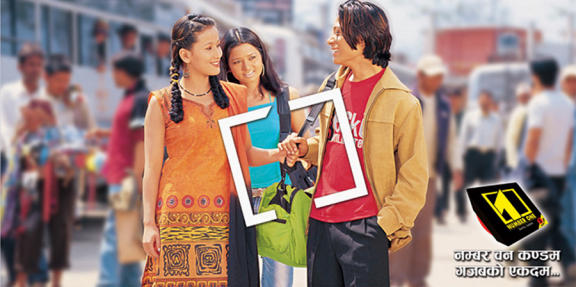
Advertisement for “Number One” Condoms Marketed in Nepal by Population Services International In April 2003, Nepal launched Number One condoms with support from USAID. In January 2004, Population Services International, a non-profit organization that uses social marketing to encourage condom use, launched Number One single-pack condoms for high-risk target groups.

## A Window of Opportunity

There is a window of opportunity to combat HIV/AIDS in Nepal. If trends continue, AIDS will be the leading cause of death among 15–49-year-olds in the next ten years [8]. 100,000 to 200,000 young adults could be infected, and 10,000 to 15,000 annual AIDS deaths could occur in the next ten years [8]. What steps must be taken to prevent Nepal from being devastated by the infection?

First, we must gather better data on HIV seroprevalence, on sexual behaviour in the general population, and on sexual networking [40]. We have argued in this essay that the extent of the AIDS epidemic in Nepal will depend upon rates at which sexual partners are exchanged by commercial sex workers and the men who regularly visit them, as well as the proportion of the general population that has multiple and concurrent sexual partners. There are extensive migration patterns both within the country and internationally, fuelled by the recent conflict, which provide the potential for considerable sexual networking. Better knowledge of this networking is crucial for our HIV control efforts.

Second, the government must recognize and acknowledge the needs of high-risk groups—drug users, commercial sex workers, migrant workers, and men who have sex with men. Plans to create more behaviour changes are needed within these groups. The 2002–2006 HIV/AIDS strategy proposed by the government [41], which adopts a multisectoral approach focusing on prevention among vulnerable groups, on control, care and support, and on voluntary counselling, hopes to address some of these issues.

Third, information campaigns should focus on changing attitudes that create barriers to regular use of condoms. Condoms should be readily available, and research should focus on the impact of mass media on perceptions of risk, negative attitudes toward condoms, and risky behaviour [27]. A culturally specific approach to HIV prevention is needed that includes education of clients and brothel owners about condoms.

Fourth, the various players involved in addressing HIV and who are working in closely adjacent fields need to interact more and partner with each other, and there needs to be greater inclusion of civil society in campaigns to raise HIV/AIDS awareness and reduce stigma.

Finally, successful national programs that have the scale to alter the course of the epidemic in Nepal should be expanded.

## Conclusion

HIV/AIDS is no longer only a health issue, it is also a development issue. Tackling the epidemic will require not only prevention and control of HIV infection among vulnerable and risk groups, but a multi-sectoral approach addressing the lack of access by risk groups to health care and education and recognition of the populations at risk [42]. People living with HIV and AIDS should be brought to the forefront in the fight against HIV/AIDS [35]. Family members, local communities, civil society organizations, donors, and the government all have their own important role to play [35]. The status of women must change so that they are considered autonomous individuals who can make their own decisions [29].

As with all international declarations on HIV/AIDS, there is an absolute need to take a strong human rights approach to combating the epidemic. This approach includes recognizing fundamental rights such as access to health care and information, addressing gender equity, and a concerted effort to reduce sex trafficking. It further requires addressing the root causes of poverty and inequality, which give rise to the phenomenon of migration and trafficking as well as propagate violent uprisings. Only such efforts will prevail in mitigating the effects of both HIV and conflict in Nepal.

## Abbreviations

IDU: intravenous drug user
NGO: non-governmental organization
